# Hematopoietic cell kinase (HCK) as a therapeutic target in immune and cancer cells

**DOI:** 10.18632/oncotarget.4199

**Published:** 2015-06-10

**Authors:** Ashleigh R. Poh, Robert J.J. O'Donoghue, Matthias Ernst

**Affiliations:** ^1^ The Walter and Eliza Hall Institute of Medical Research, Department of Medical Biology, University of Melbourne, Victoria, Australia; ^2^ Olivia Newton-John Cancer Research Institute, La Trobe University School of Cancer Medicine, Victoria, Australia

**Keywords:** SRC family kinases, hematopoietic cell kinase, cancer, leukemia, SFK inhibitors

## Abstract

The hematopoietic cell kinase (HCK) is a member of the SRC family of cytoplasmic tyrosine kinases (SFKs), and is expressed in cells of the myeloid and B-lymphocyte cell lineages. Excessive HCK activation is associated with several types of leukemia and enhances cell proliferation and survival by physical association with oncogenic fusion proteins, and with functional interactions with receptor tyrosine kinases. Elevated HCK activity is also observed in many solid malignancies, including breast and colon cancer, and correlates with decreased patient survival rates. HCK enhances the secretion of growth factors and pro-inflammatory cytokines from myeloid cells, and promotes macrophage polarization towards a wound healing and tumor-promoting alternatively activated phenotype. Within tumor associated macrophages, HCK stimulates the formation of podosomes that facilitate extracellular matrix degradation, which enhance immune and epithelial cell invasion. By virtue of functional cooperation between HCK and *bona fide* oncogenic tyrosine kinases, excessive HCK activation can also reduce drug efficacy and contribute to chemo-resistance, while genetic ablation of HCK results in minimal physiological consequences in healthy mice. Given its known crystal structure, HCK therefore provides an attractive therapeutic target to both, directly inhibit the growth of cancer cells, and indirectly curb the source of tumor-promoting changes in the tumor microenvironment.

## INTRODUCTION

**Figure 1:**
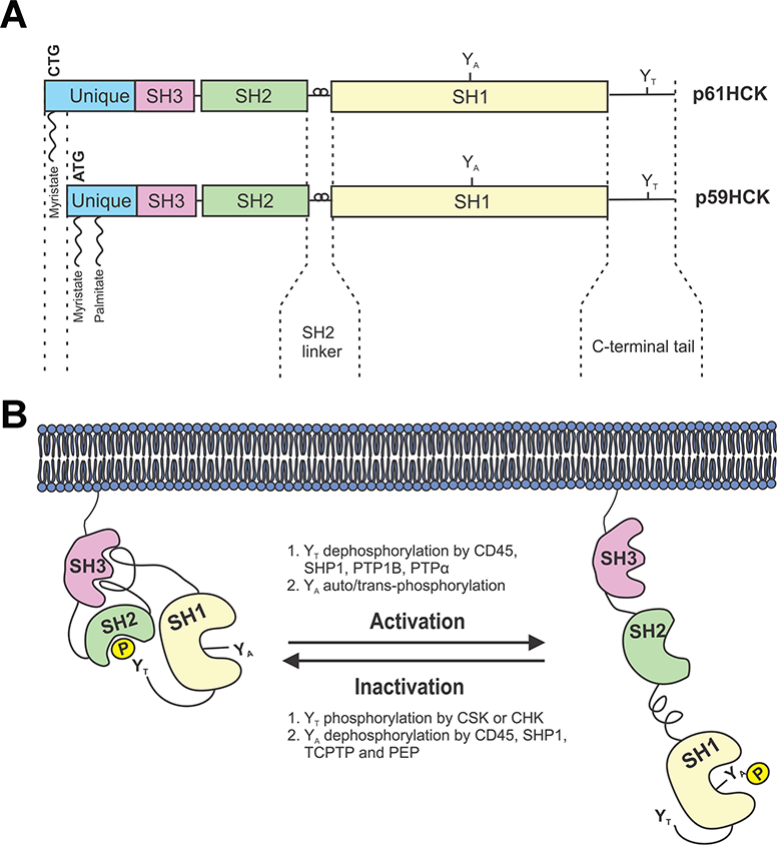
Schematic representation of HCK structure and regulation **A.** The HCK protein exists as two isoforms generated from the alternate use of two in-frame initiation codons of translation on a single mRNA. Both protein isoforms are composed of an acylated unique N-terminal domain followed by SRC Homology (SH) domains SH3, SH2 and SH1. The SH1 domain contains the catalytic domain with conserved tyrosine residues (Y_A_), while the very C-terminal tail contains a regulatory tyrosine (Y_T_). **B.** HCK is maintained in an inactive conformation by the binding of the SH2 linker to the SH3 domain, and by the binding of the phosphorylated tyrosine Y_T_ to the SH2 domain. Activation of HCK occurs following dephosphorylation of Y_T_ phosphatases, as well as auto/trans-phosphorylation of the Y_A_ residue. *Adapted from Guiet et. al, 2008*.

The SRC family of non-receptor protein tyrosine kinases (SFK) consists of 9 members, and can be divided into two groups according to the expression pattern of the individual kinases in the adult. Accordingly, SRC, YES and FYN are ubiquitously expressed, while expression of LCK, FGR, BLK, LYN, YRK and HCK is limited to certain types of cells and tissues. HCK expression is confined to cells of the myeloid and B-lymphocyte lineages and the protein exists as two isoforms. In humans, they comprise the p61HCK and p59HCK isoforms consisting of 525 and 504 amino acids, respectively, while in mice HCK exists as p59HCK (503 amino acids) and p56HCK (482 amino acids). In either species the two isoforms are generated by alternative initiation of protein translation from two in-frame initiation codons found within a single mRNA [1, 2] (Figure 1A). The two isoforms differ from each other by acylation of their N-terminal domain, and as a consequence, exhibit distinct subcellular localizations [1]. The larger isoform is mainly associated with lysosomes due to myristoylation of a glycine (Gly) residue at position +2 relative to the amino terminal methionine (Met). Meanwhile, p59HCK is mostly confined to the plasma membrane as a result of myristoylation (at Gly +2) and of palmitoylation of a cysteine or serine (at Cys/Ser +6) which form part of a Met-Gly-Cys-X-X-Cys/Ser-X consensus sequence [1, 3]. Importantly, SFK members exhibit complementary and often functionally redundant roles in several signal transduction pathways, such as the cooperation of FYN and LCK in T-cell receptor mediated activation [4, 5], and the regulation of macrophage phagocytosis by HCK and FGR [6]. This functional overlap between the SFK members has been shown to play an important role in maintaining relatively normal immune responses in situations of impairment or mutation of one of these kinases [6].

### HCK structure and regulation

HCK shares with all the other SFKs a common architecture of five distinct domains [7, 8] (Figure 1A). They comprise a SFK-family member-specific N-terminal domain required for the aforementioned lipid modifications, followed by three highly conserved SRC Homology domains (SH3, SH2 and SH1) and a C-terminal tail containing a regulatory tyrosine residue (Y_T_) at position 521 in human p61HCK, or 499 in mouse p59HCK, respectively [9]. The regulatory SH3 and SH2 domains bind to a proline-rich Pro-X-X-Pro motif and a phospho-tyrosine pTyr-Glu-Glu-Ile motif, respectively [10]. The catalytic SH1 domain contains two lobes that form the active site of the kinase, which is stabilized upon phosphorylation of a conserved tyrosine residues (Y_A_) at position 410 in human p61HCK (388 in mouse p59HCK) near the ATP binding site [9]. The small N-terminal lobe of the SH1 domain is composed of five β-sheets and a single α-helix responsible for ATP binding, while the second larger lobe is predominantly α-helical and binds to protein substrates [11].

Under steady-state conditions, HCK is maintained in an inactive conformation by two complementing intra-molecular associations (Figure 1B). The first involves binding of the SH2 domain to the phosphorylated Y_T_ at the C-terminal tail, while the second involves binding of the SH3 domain to the proline-rich type II helical motif in the linker between the SH2 and SH1 domains [10]. Two enzymatic modifications mediate this inhibitory conformation, namely dephosphorylation of pY_A_ by CD45, a trans-membrane receptor-like tyrosine phosphatase that is expressed exclusively in hematopoietic cells [12], and phosphorylation of Y_T_ by either the C-terminal SRC kinase (CSK) or the C-terminal SRC kinase-homologous kinase (CHK) [13]. In contrast to ubiquitously expressed CSK, CHK expression is limited to neurons and hematopoietic cells and may also inhibit SFKs in a non-catalytic manner by forming stable complexes with SFKs [14].

HCK activation is triggered by a variety of stimuli, including binding of bacterial lipopolysaccharide (LPS) to the CD14/Toll-like receptor (TLR)4 protein complex, cytokines such as Interleukin (IL)-2, IL6 and related ligands, and the Granulocyte Macrophage Colony Stimulating Factor (GM-CSF) receptor [15–17] (Table 1). In response to stimulation, the Y_T_ residue is dephosphorylated by CD45 or SHP1, and HCK undergoes a conformational change involving the disruption of the SH3-linker interaction which enables “unfolding” of the protein and phosphorylation of the Y_A_ residue [10]. However, HCK activation may also occur through non-catalytic mechanisms that disrupt the intra-molecular inhibitory interactions. In particular, the HIV-1 NEF protein can bind directly to the SH3 domain of HCK thereby disrupting the SH3-linker interaction and promoting a significant increase in catalytic activity [17, 18].

**Table 1:** HCK activators and substrates

Membrane associated activators	Effects	References
Beta 2 integrin	Cell adhesion	[101]
CCR3	Chemokine signaling and immune cell migration to inflammatory sites in allergic disease	[143]
CD66	Adhesion and activation of granulocytes	[144, 145]
M-CSFR and G-CSFR	Cell proliferation, differentiation, and survival	[89, 146]
FcγRI and FcγRIIa	Phagocytosis and antibody cell-mediated cytotoxicity	[147, 148]
IL2R	Cytokine production and secretion	[149]
GP130	Proliferation, cytokine production and secretion	[15, 68, 71]
uPA-R	Cell migration, adhesion and wound-healing	[150, 151]
NEF	Enhancement of viral infectivity	[17]
TLR-4	Immune surveillance	[16]
**Substrates**		
Bcr/Abl and Tel/Abl	Myeloid cell transformation and proliferation	[55, 62, 152]
CBL	Cell adhesion and transformation	[153, 154]
C3G	Apoptosis	[67]
ELMO1	Phagocytosis and cell motility	[155, 156]
GAB1 and GAB2	Cell proliferation and survival	[68]
PAG	Proliferation and transformation	[157]
Paxillin	Cell migration / podosome formation	[158]
p73	Cell cycle regulation and apoptosis	[159]
RA70	Cell differentiation	[160]
STAT5	Cell proliferation, survival and transformation	[54, 161]
VAV1	Immune cell activation, generation of reactive oxygen species and cytokine production	[162, 163]
WASP	Cell migration	[109, 155]

Upon activation, HCK engages a range of downstream signaling pathways that mediate various aspects of the immune response (Table 1). Critical roles for HCK in these processes have been demonstrated in mice carrying knock-out alleles (*HCK*^KO^) or encoding, as a knock-in mutation, a constitutively active protein with a tyrosine-to-phenylalanine (Y_499_F) substitution mutation (*HCK*^CA^). While HCK^KO^ mice display a relatively mild phenotype, macrophages from these animals exhibit reduced phagocytic ability. Consistent with functional redundancy among SFKs, compound HCK^KO^;FGR^KO^ knock-out mice show impaired myeloid cell degranulation and migration culminating in increased susceptibility to infection [6, 19]. In contrast, HCK^CA^ animals are highly responsive to LPS, and display an exaggerated innate immune response with the corresponding macrophages and neutrophils exhibiting enhanced effector functions *in vitro*, including nitric oxide and TNFα production, chemotaxis, and degranulation [20]. As a consequence, HCK^CA^ mice spontaneously acquire a lung pathology characterized by extensive eosinophilic and mononuclear cell infiltration within the lung parenchyma, alveolar airspaces, and around blood vessels, associated with epithelial mucus metaplasia, reminiscent of chronic obstructive pulmonary disorder (COPD) in humans [20].

### SFK deregulation in cancer

Deregulation of SFKs has been implicated in disease, including cancer, inflammatory and autoimmune disease [21–23]. The initial recognition of the oncogenic potential of SFKs builds on observations by the pathologist Peyton Rous linking the formation of sarcoma in chickens to (retroviral) infection of these birds [24]. Subsequently, it was shown that the genome of the Rous-sarcoma virus (RSV) encoded for a constitutive active tyrosine kinase referred to as Rous Sarcoma kinase (SRC), which transformed fibroblasts and conferred uncontrolled proliferation [25]. However, it was the seminal work by Harold Varmus and Michael Bishop in 1979, which described the discovery of a mammalian orthologue of the viral (v)-SRC protein, referred to as cellular (c)SRC. Importantly, it was shown that RSV had integrated a genomic sequence encoding for a truncated version of c-SRC that lacked the regulatory carboxy-terminal tail including the regulatory Y_T_ at amino acid position 527. Hence, unlike its cellular counterpart c-SRC, by now referred to as a “proto-oncogene”, v-SRC remained constitutively active [26]. These insights resulted in a paradigm shift for cancer not only arising from the acquisition of foreign DNA (i.e. viruses), but more commonly by acquiring mutations in endogenous genes that transformed highly regulated proto-oncogenes to *bona fide* oncogenes encoding proteins permanently locked in their active conformation. Elevated c-SRC activity and mutations that functionally replicate the v-SRC truncation have now been detected in a wide variety of malignancies. While the oncogenic potential of c-SRC is widely accepted, we discuss here emerging novel mechanisms by which deregulated HCK activity contributes to tumor initiation and progression, including gene amplification and the interaction with regulatory phosphatases and upstream receptor tyrosine kinases.

#### SFK overexpression

Increased SFK activity and/or gene expression is frequently detected in tumor biopsies [27]. *HCK* gene amplification is observed in poorly differentiated human gastric cancer [28] and several colorectal cancer cell lines [29]. Increased *SRC* expression is observed in intestinal adenomas of the *Apc^Min^* mouse model of familial adenomatous polyposis, suggesting that the WNT/β-catenin pathway may contribute to increased SRC signaling [30]. Epigenetic changes have also been proposed to induce overexpression of SFKs [21]. Furthermore, microRNA-23b and miRNA-145, which are downregulated in many prostate and colorectal cancers, repress SRC and YES activity [31, 32]. These observations collectively suggest that SFK overexpression in tumor cells can occur as a result of exaggerated activation of upstream signaling pathways as well as aberrant epigenetic modifications to exaggerate SFK activation.

#### SFK activation by suppression of CSK and Cbp/PAG

Phosphorylation of Y_T_ is crucial for SFK inactivation and the loss of this residue confers constitutive activation and transforming capabilities of oncogenic v-SRC and v-YES proteins [33]. Disruption of the interaction between Y_T_ and the SH2 domain can result from impaired CSK activity or from pY-residues of growth factor receptors, which compete for binding to the SH2 domain in SFKs, thereby yielding an open and catalytically active conformation [34]. Although CSK was initially thought to act as a tumor suppressor, its contribution to cancer remains less clear. In hepatocellular carcinoma, reduced CSK levels inversely correlate with SFK activity [35]. However, increased CSK expression along with SFK activity has been observed in primary carcinoma [36] and human colorectal cancer cell lines [37], indicating that CSK-dependent regulation of SFK signaling may be specific to some cancers. CSK may also contribute to SFK deregulation by altered trafficking, since CSK-mediated inhibition of SFK relies on membrane-associated CSK binding proteins [38]. Interestingly, this recruitment step is impaired in colorectal cancer, resulting in retention of CSK in the cytoplasm [39]. In accordance with these observations, expression of Cbp/PAG, which traffics CSK to the membrane, is frequently reduced in human colorectal tumors, while introduction of Cbp/PAG into metastatic colorectal cancer cells restores membrane translocation of CSK and reduces invasion of these cells into extracellular matrix [39]. Collectively, these results suggest an important role for Cbp/PAG in ensuring proper CSK localization and associated physiological regulation of SFK activity.

#### SFK activation by tyrosine phosphatases

SFKs can also act as substrates for protein tyrosine phosphatases other than CD45 and SHP1, which otherwise primarily regulate the activity of SFKs. Increased activity of PTPα, PTPB1, TCPTP and other tyrosine phosphatases may also contribute to elevated SFK activity and sustained signaling [40]. Short hairpin RNA-mediated suppression of PTPα, for instance, reduced SRC activity by up to four-fold in human breast and colon cancer cell lines [41], while overexpression of PTP1B increased SRC activity and promoted anchorage-dependent growth of colon cancer cells [42]. In addition, inhibition of tyrosine phosphatases may also promote SFK activation by increasing the level of the phosphorylated Y_A_ residue in their SH1 domain. TCPTP protein levels are reduced in a subset of estrogen receptor, progesterone receptor and HER2 triple-negative human breast cancers, and *TCPTP* deficient breast cancer cell lines exhibit elevated SFK signaling [43]. Conversely, reintroduction of TCPTP into these cells reduced SFK activity, and impaired cell proliferation and anchorage-independent growth [43]. Likewise, treatment of pancreatic cancer cells with tyrosine phosphatase inhibitors increased pY_A_ of SRC in adherent cells [44], while PTPL1 inactivation in colorectal cancer cells promoted anchorage-independent cell growth [45]. Together, these results suggest that inhibition of tyrosine phosphatases plays a key role in mediating excessive SFK activity in cancer cells.

#### SFK association with receptor tyrosine kinases

**Figure 2:**
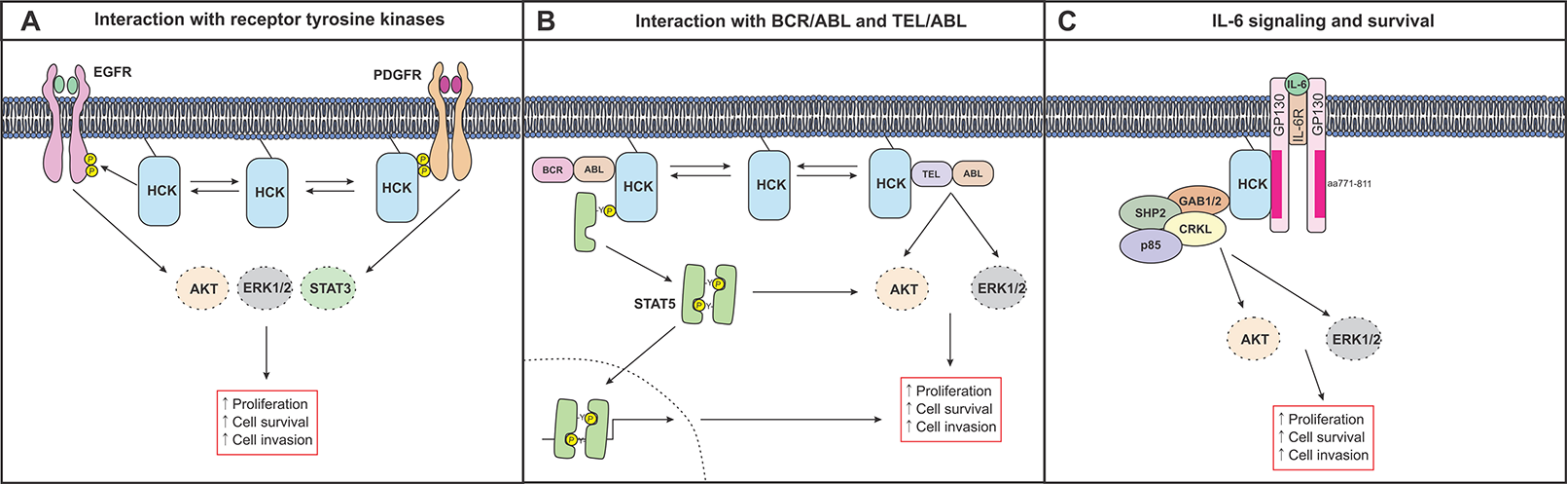
Simplified schematic depiction of HCK signaling in cancer cells **A.** The interaction of HCK with receptor tyrosine kinases such as EGFR and PDGFR results in the activation of ERK, AKT and STAT3 signaling pathways to promote cell proliferation in cancer cells. **B.** Direct association of HCK with BCR/ABL results in the persistent activation of STAT5 and its retention in the cytoplasm. Within the cytoplasm, STAT5 promotes cell growth and survival through the activation of AKT. HCK can also interact with TEL/ABL to promote proliferation, survival and invasion through the activation of AKT and ERK pathways. **C.** HCK can bind directly to an acidic domain (comprising of amino acids 711–811) of GP130 and mediate ERK and AKT signaling to induce cell survival and proliferation in response to IL6 and related cytokines.

The association of SFKs with receptor tyrosine kinases (RTK) has been implicated in situations of persistent RTK signaling in cancer [46]. SFK activation occurs in response to ligand binding of platelet-derived growth factor receptor (PGDFR), epidermal growth factor receptor (EGFR), fibroblast growth factor receptor (FGFR) and others. In these situations, SFKs are involved in the transduction of signals required for receptor turnover, actin cytoskeletal rearrangement and cell survival [46]. There is evidence that the SH2 domain of SFKs can bind directly to pY599 in PDGFR [47] (Figure 2A). This interaction disrupts intramolecular interaction between the SH2 domain and the pY_T_ residue in SFKs, and exposes their Y_A_ residue to become available for phosphorylation [34]. SFK activation can also occur by an indirect mechanism, since EGFR does not contain consensus pY residues to enable binding of the SH2 domain of SFKs (Figure 2A). In epithelial cancers expression of EGFR, ERBB2 and to a lesser extent other EGFR-family members, is increased by mutation and/or gene amplification and this is commonly associated with excessive SRC signaling [48, 49]. Indeed, excessive SFK activity can activate these RTKs in the absence of their ligands [39]. Meanwhile, excessive activation of c-MET, EPHA2 and other RTKs is required for maintaining maximal SRC activity in colorectal cancer cells [50], suggesting that SFK deregulation may create a feedback loop whereby SFK activity increases with RTK activity to fuel tumor promotion.

### HCK signaling in leukemia

HCK expression is elevated in various types of leukemia (Table 2), and contributes to leukemogenesis through its association with the oncogenic fusion proteins BCR/ABL and TEL/ABL to enhance cell survival. However, decreased HCK activation has also been reported in a small number of acute myeloid leukemia and acute promyelocytic leukemia patients.

**Table 2:** HCK expression in various forms of leukemia

Type	HCK expression	HCK associated cellular effects	References
**Chronic myeloid leukemia**	Elevated	Cellular transformation	[161, 164]
**Acute myeloid leukemia** *inv(16)(p13q22) and Philadelphia chromosome negative cases*	Reduced	Downregulated apoptosis	[63, 65]
**Acute promyelocytic leukemia**	Reduced	Downregulated apoptosis, increased survival	[65]
**Multiple myeloma**	Elevated	Excessive IL6 signaling and cell proliferation and survival	[68, 70]
**Acute lymphoblastic leukemia**	Elevated	Cell proliferation and survival	[62, 165]

#### Interaction with oncogenic fusion proteins

Chronic myeloid leukemia (CML) accounts for 20% of leukemias and is characterized by excessive production of granulocytes that accumulate in the bone-marrow and interfere with normal hematopoiesis. Over time, leukemic cells build up and spill into the bloodstream, eventually accumulating in organs and impairing the normal functions of immune cells. HCK has been implicated in CML through its direct association with, and activation by BCL/ABL [51] (Figure 2B). The BCR/ABL oncogenic fusion protein is derived from translocation of the *c-ABL* gene on chromosome 9 to the *BCR* locus on chromosome 22 and is present in a large majority of CML cases, as well as in some acute lymphocytic leukemias [52, 53]. In response to its association with BCR/ABL, HCK mediates persistent activation of STAT5A and the excessive accumulation of this latent transcription factor in the cytoplasm [51], where STAT5A engages PI3K and the adaptor protein GAB2 to activate AKT and to facilitate cell growth and survival [51]. Interestingly, despite the functional redundancy among SFK members, HCK appears to be the most important kinase in BCR/ABL-induced signaling [51, 54]. Overexpression studies demonstrate a physical interaction between HCK and STAT5A that culminates in STAT5A phosphorylation. While similar experiments showed that LYN was also capable of inducing STAT5A phosphorylation, there was no evidence of physical interaction between LYN and STAT5A [54]. Furthermore, co-expression of kinase-defective HCK mutants with BCR/ABL strongly impaired transformation of cytokine-independent growth of myeloid leukemia cells [55], while treatment with the broad-spectrum SFK inhibitor PP2 abrogated BCR/ABL-dependent activation of STAT5A [54]. These studies identify a dominant role for HCK in mediating BCR/ABL-induced activation of STAT5.

Expression of the TEL/ABL oncogenic fusion protein is observed in T- and pre-B cell acute lymphoblastic leukemias and some CML subsets [56–58], and promotes constitutive activation of STAT5, ERK, AKT and other pathways to facilitate the growth and survival of cancer cells [59–61]. HCK activation is increased in TEL/ABL-expressing Ba/F3 cells. Conversely treatment with Imatinib, which also suppresses TEL/ABL activity, inhibits HCK activation as well as STAT5, ERK and AKT signaling [62] (Figure 2B). Likewise, treatment of these cells with the SFK inhibitors PP1, PP2 or SU6656 abrogates TEL/ABL activity and induces growth arrest [62]. Indeed, HCK appears to be a critical regulator of TEL/ABL-dependent growth, since expression of kinase-dead HCK mutants inhibits the growth of TEL/ABL transformed cells and the phosphorylation of ERK1/2 and AKT [62].

Although HCK expression is frequently increased in acute and chronic myeloid leukemia, HCK activity is reduced in acute myeloid leukemia patients with the specific chromosomal rearrangement inv(16)(p13q22) [63]. Likewise, in acute lymphocytic leukemia patients, which lack the BCR/ABL fusion protein, HCK expression is reduced due to aberrant *HCK* gene methylation [64], while in acute promyelocytic leukemia, *HCK* transcription is repressed by the fusion protein promyelocytic leukemia-retinoic acid receptor α (PML-RARα). Since the PML-RARα protein promotes survival of myeloid precursor cells, it has been proposed that HCK inhibition may facilitate tumorigenesis by interfering with apoptosis [65]. In line with this, knock-down of HCK by siRNA substantially reduced expression of the anti-apoptotic protein MCL-1, which coincided with increased expression of pro-apoptotic BAX and enhanced death of cancer cells [66]. Meanwhile, the interaction of HCK with the guanine nucleotide exchange factor C3G was shown to indirectly induce apoptosis via a Caspase-1/8/9-dependent mechanism, which depended on the catalytic activity of HCK [67]. However, since the latter observation arose from overexpression in epithelial cells, its physiological relevance remains unclear.

#### IL6 signaling and survival

Multiple myelomas are characterized by excessive production of plasma cells in the bone-marrow and require IL6 signaling to promote cell growth and survival through activation of the ERK and PI3K signaling cascade [68]. Ligation of IL6 to its receptor results in juxta-positioning of the two IL6R β-chains (also referred to as GP130) and activation of the associated JAK kinases, which in turn confers phosphorylation of conserved cytoplasmic tyrosine residues in GP130. They comprise the membrane proximal pY that provides a docking site for the tyrosine phosphatase SHP2 and the negative regulator SOCS3, and four membrane distal pY, which serve as binding sites for STAT3 and STAT1 [69]. In multiple myeloma cells, various investigators have identified FYN, HCK and LYN in co-immunoprecipitates with GP130, and stimulation of these cells with IL6 results in increased activation of these SFKs [70]. However, the interaction between HCK and GP130 may be functionally more important for mediating proliferative signals than binding of FYN or LYN to GP130 [70]. Surprisingly, HCK was shown to mediate ERK signaling independently of the aforementioned association between GP130 and STAT3 or SHP2, but rather through direct binding of HCK to an acidic domain in GP130 (Figure 2C). Furthermore, deletion of this region (amino acids 771–811) impaired IL6 dependent HCK activation and reduced cell proliferation [71]. In line with this, expression of a kinase-dead HCK mutant in mouse myeloma 7TD1 cells elicited a dominant-negative effect on cell proliferation, thereby providing additional evidence that HCK is required to transmit GP130-dependent growth signals [72]. Similarly, we reported a functional contribution of HCK to the GP130-mediated signals that retain pluripotency of murine embryonic stem cells [15], where HCK undergoes rapid and transient activation in response to cells stimulated with the GP130 ligand leukemia inhibitory factor, LIF [73]. As in multiple myeloma cells, we also found that HCK directly associates with GP130 in embryonic stem cells [15].

The mechanism by which the GP130/HCK-containing complex promotes IL6-induced activation of ERK and PI3K in multiple myeloma cells is dependent on the adaptor proteins GAB1 and GAB2, which are constitutively associated with HCK. These scaffolding proteins are involved in the recruitment of multimeric protein complexes containing SHP2, GRB2, CRKL and the p85 subunit of PI3K, which promote activation of ERK and other downstream signaling molecules [68]. Importantly, kinase-dead HCK mutants, or treatment with the SFK inhibitor PP2, reduced IL6-induced tyrosine phosphorylation of intracellular signaling proteins and significantly decreased myeloma cell survival and proliferation [68]. Together, these results implicate HCK, and possibly its association with the GAB adaptor proteins, as key players in GP130-mediated multiple myeloma pathogenesis.

### HCK activation in tumor-associated immune cells

Excessive HCK expression is observed in various solid cancers, including those of the breast [16, 74]. The microenvironment of solid cancers comprises a heterogeneous population of stromal cells that include fibroblasts, endothelial cells, adipocytes, mesenchymal cells and immune cells. All of these cells “communicate” with cancer cells and each other to promote tumor development and progression [75] through the secretion of growth factors [76] and cytokines [77] that promote survival, angiogenesis [78], invasion [79] and metastasis [80, 81]. Stromal cells also play a key role in suppressing anti-tumor immune cell responses and conferring resistance to chemotherapy [82, 83].

**Figure 3:**
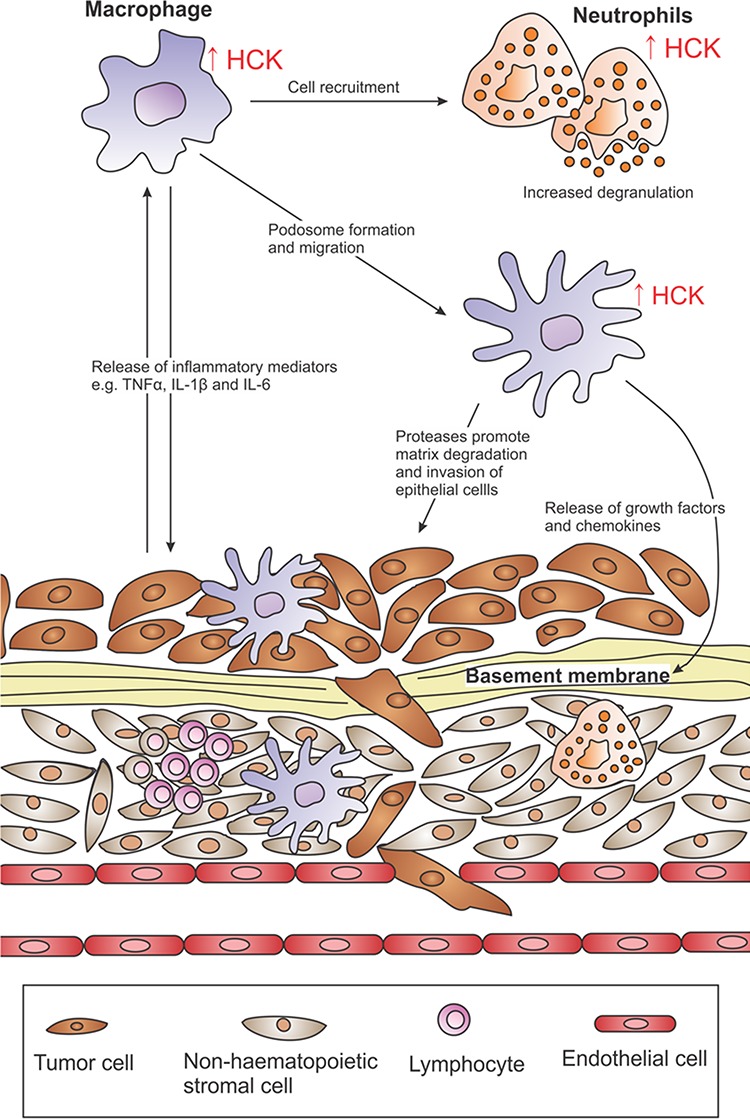
HCK activation in the tumor microenvironment Excessive HCK activation (↑ HCK) is a common observation in many solid cancers and enhances the recruitment of immune cells into tumors and promotes their proliferative and migratory ability. HCK is also involved in inflammatory signaling and its expression augments the release of TNFα, IL1β and IL6 from macrophages. These cytokines can feedback onto tumor cells to perpetuate inflammation and sustain neoplastic transformation. HCK activation plays a key role in podosome formation in macrophages and promotes their phagocytotic, chemotactic and matrix degradation abilities. This may enhance immune and epithelial cell invasion by facilitating large-scale degradation of the extracellular matrix.

Myeloid cells, including macrophages, myeloid-derived suppressor cells and neutrophils make up a major component of the stromal compartment and contribute to the tumor promoting mechanisms described above [84]. In particular, tumor-associated macrophages (TAMs) comprise distinct subsets of alternatively activated macrophage that orchestrate many of the aforementioned hallmarks and enabling characteristics of cancer. Likewise, tumor-associated neutrophils have been shown to facilitate and promote the suppression of immune cell mediated anti-tumor responses [85]. HCK plays a key role in neutrophil phagocytosis [86, 87], and has been shown to co-localize with β-arrestin on the G-protein-coupled IL8 receptor, where HCK traffics to azurophilic granule-rich regions to initiate the release of their content [88]. Similarly, HCK also regulates macrophage activity by promoting their proliferation [89], migration [90] and secretion of IL6, TNFα and other cytokines [16, 91]. Thus, in addition to its direct oncogenic role in (leukemic) cancer cells, excessive HCK activation may be stimulated in neighboring immune cells by incipient tumor cells through cytokine secretion thereby reinforcing a tumor-enabling and tumor-promoting microenvironment (Figure 3).

#### Inflammation

It has been recognized for more than a century that inflammation is an enabling characteristic of many cancers, and increased expression of pro-inflammatory cytokines, chemokines, prostaglandins and associated enzymes are frequently detected in human and murine tumors [92]. In colorectal cancer, for instance, TNFα [93], IL6 [94] and IL1β [95] from tumor infiltrating neutrophils has been shown to assist in neoplastic transformation and expansion of the mutated epithelium. Furthermore, chronic inflammation associated with hepatitis C virus infection results in excessive TGFβ signaling, which promotes cirrhosis and hepatocellular carcinoma [96], while long-term cigarette smoke exposure increases the risk of lung cancer and exacerbates lung inflammation [97]. Neoplastic cells secrete inflammatory molecules such as TNFα [98] and IL1β [99], likely as part of a wound-healing response conserved from their epithelial origins thereby forming a paracrine loop which perpetuates inflammation and sustain the activity of inflammatory immune cells.

HCK is involved in inflammatory signaling, and in human macrophages its increased expression augments TLR4-induced transcription of TNFα and IL6 via the AP-1 transcription factor complex [16, 100]. Consistent with this, genetic ablation of *HCK* in mice reduced cytokine expression in response to LPS stimulation, and moderately increased the susceptibility of HCK^KO^ mice to infection as a consequence of impaired immunity [6]. Conversely, HCK^CA^ mice display hypersensitivity to LPS-dependent endotoxemia associated with excessive secretion of TNFα and other inflammatory mediators [20]. HCK activation is also critical in integrin-dependent adhesion and immune cell recruitment during systemic inflammation [101, 102]. Thus, HCK provides a critical node that links the extracellular environment to an adequate innate immune cell response characterized by cytokine release and local immune cell recruitment.

Although a functional link between HCK, inflammation and solid cancers still remains somewhat tenuous, we have observed that a small proportion of aging HCK^CA^ mice develop lung adenocarcinomas as a subsequence to their spontaneous lung inflammation with characteristics of COPD [20]. In humans, COPD is a major risk factor for lung cancer and is characterized by increased numbers and activity of polymorphonuclear leukocyte in sputum and bronchial lavage fluid of these patients [103]. Consistent with this, HCK protein levels are significantly elevated in neutrophils from COPD patients [104]. Furthermore, genomic amplification of the *HCK* locus has been observed in non-small cell lung cancer [105, 106], suggesting a potential role for HCK in promoting COPD-associated inflammation and predisposition to lung cancer.

#### Migration and invasion

Metastatic spread is the major cause for cancer mortality and occurs when cancer cells dissociate from the primary tumor and seed into distal organs. Highly invasive cancer cells form specialized matrix-degrading membrane protrusions known as invadopodia, which enable cancer cells to migrate through the tumor stroma and to intravasate into blood and lymphatic vessels, from where the cancer cells can spread to distant sites. TAMs form protrusions known as podosomes that are similar to invadopodia in molecular composition and proteolytic activity [107]. Thus, the ECM-remodeling ability of podosomes by TAMs could increase tumor invasiveness by assisting the formation of tunnels in the matrix, thereby enabling the escape of tumor cells independent of their own matrix degrading abilities.

HCK plays a crucial role in podosome formation by for instance phosphorylating the Wiskott-Aldrich syndrome protein (WASP), a hematopoietic cell-specific and actin nucleation promoting factor that regulates actin polymerization and promotes phagocytosis, chemotaxis, podosome formation and matrix degradation [108, 109]. In bone marrow-derived macrophages from HCK^KO^ mice, and in human HCK knock-down macrophage cell lines, tyrosine phosphorylation of WASP in response to CX3CL1 or Fcγ stimulation is significantly impaired, and correlates with reduced phagocytosis, chemotaxis and matrix degradation by these cells [109]. Individual overexpression of the HCK isoforms in murine fibroblasts revealed that p59HCK^CA^ triggered the formation of plasma membrane protrusions, while p61HCK^CA^ resulted in podosomal formation [110]. Importantly, co-expression of both isoforms conferred a transformed phenotype in these cells, manifested by the loss of contact inhibition, shortened cell doubling time and serum independent growth.

Stimulation of macrophages with LPS and/or IFNγ, or with macrophage-colony stimulating factor not only activates HCK, but also promotes formation of podosomes and their arrangement into rosette clusters [111, 112]. Indeed, HCK^KO^ macrophages exhibit reduced rosette forming ability and impaired matrix degradation, consistent with reduced macrophage recruitment and decreased efficiency in promoting invasion of tumor cells [90, 113]. These findings suggest that HCK is involved in podosomal formation and ECM-degradation by macrophages, which may enhance immune and epithelial cell invasion and therefore comprise a key step during tumor progression. Thus, while HCK has a direct role in promoting some blood-derived cancers, its aberrant activation in innate immune cells of the tumor microenvironment facilitates tumorigenesis and enables progression in experimental models and in human cancers.

### HCK is associated with reduced chemo-sensitivity and acquired resistance

Reduced sensitivity and resistance to chemotherapy is a major cause of tumor recurrence, and may arise from genetic mutations, amplifications or epigenetic changes that increase the expression or activity of pro-survival factors, or regulate the uptake, metabolism and export of cytotoxic drugs [114]. In the case of breast cancer, tumor cells have been shown to recruit and stimulate macrophages to secrete the TLR4 ligands S100A8/9 thereby reducing chemotherapy-induced apoptosis [115]. Although HCK participates in the transduction of TLR4-dependent signals, this study did not investigate HCK activity in recruited macrophages. However, HCK activity either alone or within the context of other SFKs often reduces the efficacy of recombinant immunotoxins [66], where antibody fragments are tethered to an exotoxin that induces apoptosis upon antigene binding to the surface of tumor cells [116]. Accordingly, co-treatment of cells with the recombinant immunotoxin SS1P and the SFK inhibitor Bosutinib synergistically inhibited the growth of A431/H9 human epithelial carcinoma cells, and also enhanced regression of the corresponding tumor xenografts in mice [66].

### HCK may be associated with reduced drug efficacy in the clinic

The mechanism of action of most small molecule kinase inhibitors depends on their binding into the hydrophobic pocket adjacent to the ATP binding site [117]. However, the accessibility of small molecule inhibitors is often contingent on the presence of a small amino acid (e.g. threonine) adjacent to the hydrophobic pocket. Tyrosine kinase mutants that possess larger amino acids (e.g. methionine) at this position can confer clinical resistance, as exemplified in acquired resistance to Imatinib, Gefitinib or Erlotinib [118–121]. Thus, expression of a HCK threonine-to-methionine mutant in BCR/ABL transformed myeloid cells confers resistance against the apoptotic and anti-proliferative effects of the broad-spectrum SFK inhibitor A-419259 [118]. Since HCK also interacts with the BCR/ABL fusion protein, this may have clinical relevance, whereby Imatinib can no longer bind to and inhibit the BCR/ABL fusion protein in CML patients [55, 122].

HCK, alongside other SFKs, can also interfere with drug efficacy by cooperating with receptor kinases that play a major role in oncogenesis. Increased EGFR expression is observed in many human malignancies of epithelial origin, including colorectal cancer, non-small cell lung cancer and brain cancer [123]. EGFR is partially activated upon ligand binding and phosphorylation of its C-terminal tail, but requires interaction with SFKs for full activation. This induces a conformational change in the SFK, resulting in its auto-phosphorylation and transient activation [124]. In turn, SFKs phosphorylate EGFR on an additional tyrosine (Y_845_), resulting in EGFR-mediated proliferation of breast cancer cells via STAT5B [125]. Increased SFK activity is also observed in the NCI-H226 human lung squamous carcinoma cell line with acquired resistance to the EGFR-inhibitor Cetuximab, and SFK activity has been shown to cooperate with EGFR to enhance survival via HER3 and PI3K/AKT signaling [123]. Consistent with this, treatment of Cetuximab-resistant cells with the SFK kinase and BCR/ABL inhibitor Dasatinib restored Cetuximab sensitivity, while simultaneous inhibition of SFKs and EGFR markedly suppressed HER3 and PI3K/AKT signaling [123]. Together, these findings suggest that SFKs and EGFR cooperate in acquired resistance to Cetuximab, providing a strong rationale for combinatorial strategies against SFKs and EGFR in the treatment of cancer.

### Co-targeting SFKs in the clinic

Due to the prevalence of excessive SFK expression in many human malignancies and its correlation with a poorer prognosis, these kinases represent a readily actionable therapeutic target for the treatment of cancer. Several ATP-competitive kinase inhibitors with significant activity against SFKs have been developed (Table 3) [126–128]. However, preclinical tests have revealed that the efficacy of these drugs on primary tumors is variable and dependent on the level of SFK deregulation [29, 129, 130].

**Table 3:** Anti-tumor activities of SFK inhibitors

Drug	Examples of targets	Activities on tumor cells				Clinical trials
		Proliferation	Apoptosis	Invasion, Metastasis	Drug re-sensitivity	
Bosutinib	SFKs, ABL, CAMK2G, TEC, STE20	Reduced [166, 167]	Increased [168]	Reduced [169, 170]	Restored [171]	CML [172] and breast cancer [173]
Dasatinib	SFKs, BCR/ABL, c-Kit, Ephrins, RTKs	Reduced [174–177]	Increased [178–180]	Reduced [181, 182]	Restored [123, 134]	Prostate [183] and breast [184, 185] cancer
Saracatinib	SFKs, BCR/ABL, EGFR	Reduced [186, 187]	Increased [132]	Reduced [188, 189]	Restored [190, 191]	Melanoma [192] and lung [193] cancer
PP2	SFKs, RIP2, CK1δ	Reduced [194]	Increased [195]	Reduced [196]	Restored [197]	Not tested
A-419259	SFKs	Reduced [118, 198]	Increased [118]	Reduced [198]	Not tested	Not tested
RK-20449	HCK	Reduced [142]	Increased [142]	Not tested	Not tested	Not tested
SU6656	SFKs, PGDF, BRSK2, AMPK, Aurora C, Aurora B, CaMKKβ	Reduced [199]	Increased [200]	Reduced [201]	Restored [66]	Not tested

Given the various roles of SFKs in cells of the tumor stroma, SFK inhibitors have exhibited a great impact in slowing tumor invasion and metastasis. For example, combining the SFK and ABL inhibitor Saracatinib with a MEK inhibitor prevented melanoma cell invasion [131] and the metastatic outgrowth of dormant tumor cells [132]. Likewise, the synergistic activity of Dasatinib with Oxaliplatin promoted shrinkage of metastatic nodules as a consequence of reduced proliferation and angiogenesis [133]. Furthermore, Dasatinib treatment can restore the aforementioned Cetuximab sensitivity of EGFR-positive breast cancers, and also overcome BRAF inhibitor resistance in melanoma cells [134]. It is therefore likely that SFK inhibition may also provide benefit as adjuvant therapy with current standard of care chemotherapies.

### Future perspectives

To maximize the therapeutic benefit from incorporating HCK (or SFK) inhibition into existing cancer treatments, several challenges need to be addressed. One major limitation is the lack of appropriate biomarkers to monitor SFK deregulation in patients. This is crucial, since the therapeutic efficacy of SFK inhibitors will be influenced by the extent of SFK activity in tumors [29], as well as other molecular alterations which may impact on the response of cancer cells to SFK inhibitors [135]. To date, preclinical trials using SFK inhibitors have been performed in unselected patients with relatively low overall clinical benefits. However, favorable responses have been observed when patients were stratified, which emphasizes the importance of utilizing predictive biomarkers to identify those individuals who will benefit the most from SFK inhibitor therapy [136, 137].

Secondly, although SFK inhibition is effective in some advanced tumors, particularly in combination with treatments that target other tumor drivers such as HER2, other clinical trials have reported limited benefits [138–140]. For this reason, the use of SFK inhibitors in an early adjuvant setting may be a more effective in delaying the progression of emerging tumors rather than to treat advanced/metastatic disease, since a significant impact of many SFK inhibitors was associated with their capacity to slow down tumor invasion and metastasis [131, 133].

Lastly, new strategies for the design of more conformation specific SFK inhibitors are required, since drugs with high specificity and potency are more effective and exhibit fewer side effects and toxicity. In particular, proteomic tools that interrogate kinase active sites provide a powerful approach for studying protein regulation and for performing screens for highly selective inhibitors for components of complex proteomes [141]. One such new technique involves the use of probes that harbor complimentary functionalities, including distinct moieties to direct interaction to the active site, to facilitate covalent binding once docked at the kinase active site, and to provide chemo-selective tags for reporter conjugation. When applied to HCK, this screening strategies enabled rapid profiling of novel active sites in the kinase domain and the identification of a number of conformation-specific ATP competitive inhibitors [141]. An other approach exploited a library of pyrazolo[3, 4-*d*]pyrimidine derivatives to identify candidates, which bind to the ATP binding site of HCK [11]. These new ATP-competitive inhibitors have structures distinct from any class of existing HCK inhibitors and showed significant inhibitory and anti-proliferative effects at sub-micromolar concentrations [11]. Other emerging SFK inhibitors with high specificity against HCK include A-419259 [118], which blocks proliferation and induces apoptosis in CML cell lines, as well as RK-20449 [142], which promotes tumor regression in a mouse xenograft model.

## CONCLUSIONS

An oncogenic role of HCK has been implicated in human cancers, including leukemia and solid tumors of the breast, colon and stomach. Given the high frequency of excessive HCK activation in these cancers and its correlation with a poor prognosis on one hand, and the relatively mild phenotype displayed by HCK^KO^ mice on the other hand, HCK may be a promising target for cancer treatment. In particular, therapeutic-targeting strategies aimed at inhibiting HCK within the tumor stroma may be part of a paradigm-shifting approach to treating human cancers in the future. Given the role of stromal cells as key enablers and enhancers of many of the hallmarks of cancer, we predict that the inhibition of HCK in tumor-associated innate immune cells will reduce their ability to promote angiogenesis, inflammation, migration and invasion. This will be aided by the emergence of new strategies to identify novel HCK inhibitors with greater specificity and potency, which will not only provide additional insights into the mechanistic by which HCK promotes the progression of tumors, but facilitate translational outcomes for cancer patients in the clinic.
